# 5-(Chloro­meth­yl)quinolin-8-yl acetate

**DOI:** 10.1107/S1600536808021788

**Published:** 2008-07-19

**Authors:** Ling-Qian Kong, Yan Qiao, Ji-Dong Zhang

**Affiliations:** a Dongchang College, Liaocheng University, Liaocheng 250059, People’s Republic of China

## Abstract

The title compound, C_12_H_10_ClNO_2_, crystallizes with two independent mol­ecules in the asymmetric unit; these are approximate mirror images of each other. In each mol­ecule, the chloro­methyl and acetate groups lie on the same side of the quinoline ring system, with dihedral angles between the ring plane and the plane of the acetate group of 82.0 (1) and −79.2 (1)°. The C—C—C—Cl torsion angles for the chloro­methyl groups of the two mol­ecules are 80.9 (2) and −83.1 (2)°.

## Related literature

For related literature, see: Chen & Shi (1998 ▶); Marian (1966 ▶).
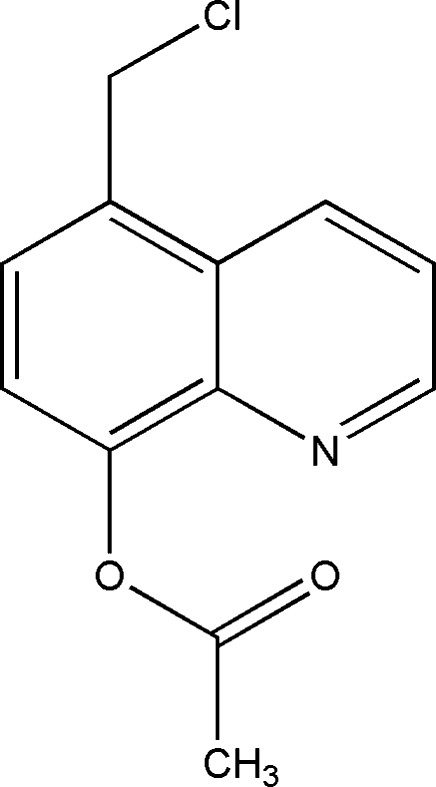


## Experimental

### 

#### Crystal data

C_12_H_10_ClNO_2_
*M* *_r_* = 235.66
Triclinic, 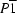

*a* = 9.2299 (10) Å
*b* = 11.0042 (13) Å
*c* = 11.2429 (13) Å
α = 105.073 (4)°
β = 94.105 (1)°
γ = 90.815 (2)°
*V* = 1099.2 (2) Å^3^
*Z* = 4
Mo *K*α radiation
μ = 0.33 mm^−1^
*T* = 295 K
0.22 × 0.18 × 0.16 mm

#### Data collection

Bruker SMART CCD diffractometer
Absorption correction: multi-scan (*SADABS*; Sheldrick, 1996 ▶) *T*_min_ = 0.931, *T*_max_ = 0.949
5721 measured reflections
3843 independent reflections
3247 reflections with *I* > 2σ(*I*)
*R*_int_ = 0.015

#### Refinement

*R*[*F* ^2^ > 2σ(*F* ^2^)] = 0.033
*wR*(*F* ^2^) = 0.096
*S* = 1.04
3843 reflections
289 parameters
H-atom parameters constrained
Δρ_max_ = 0.19 e Å^−3^
Δρ_min_ = −0.20 e Å^−3^

### 

Data collection: *SMART* (Siemens, 1996 ▶); cell refinement: *SAINT* (Siemens, 1996 ▶); data reduction: *SAINT*; program(s) used to solve structure: *SHELXS97* (Sheldrick, 2008 ▶); program(s) used to refine structure: *SHELXL97* (Sheldrick, 2008 ▶); molecular graphics: *SHELXTL* (Sheldrick, 2008 ▶); software used to prepare material for publication: *SHELXTL*.

## Supplementary Material

Crystal structure: contains datablocks global, I. DOI: 10.1107/S1600536808021788/bi2295sup1.cif

Structure factors: contains datablocks I. DOI: 10.1107/S1600536808021788/bi2295Isup2.hkl

Additional supplementary materials:  crystallographic information; 3D view; checkCIF report

## Figures and Tables

**Fig. 1.**
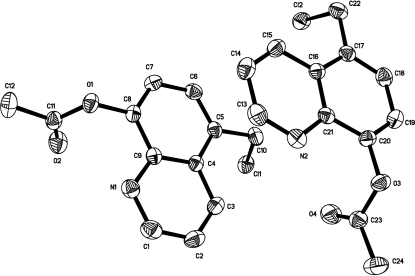
Two independent molecules in the title compound, with displacement ellipsoids drawn at the 50% probability level. H atoms have been omitted.

## supplementary crystallographic information

### Comment

8-Hydroxyquinoline and its derivatives are amongst the most extensively investigated ligands in coordination chemistry (Chen & Shi, 1998). In the course of our studies on 8-hydroxyquinoline derivatives, we have synthesised the title compound, which is a key intermediate in the synthesis of 8-hydroxyquinoline derivatives.

### Experimental

5-(Chloromethyl)quinolin-8-ol hydrochloride (0.0217 mol) (Marian, 1966) and acetic anhydride (25 ml) were added to a 100 ml flask, and refluxed for 6 h. After cooling to room temperature, the mixture was poured into cool water (500 ml). The precipitate was washed with a large amount of water, collected by filtration, and dried to produce the title compound as a grey solid. Colourless single crystals suitable for X-ray diffraction analysis were obtained by slow evaporation of an ethanol solution over a period of 2 d.

### Refinement

All H atoms were placed in geometrically idealized positions with C(*sp*^2^)—H = 0.93, C(methyl)—H = 0.96, and C(methylene)—H = 0.97 Å and constrained to ride on their parent atoms, with *U*_iso_(H) = 1.2*U*_eq_(C) (or 1.5*U*_eq_ for methyl H).

### Crystal data

**Table:**

C_12_H_10_ClNO_2_	*Z* = 4
*M**_r_* = 235.66	*F*_000_ = 488
Triclinic, *P*1	*D*_x_ = 1.424 Mg m^−^^3^
Hall symbol: -P 1	Melting point: 400 K
*a* = 9.2299 (10) Å	Mo *K*α radiation λ = 0.71073 Å
*b* = 11.0042 (13) Å	Cell parameters from 3502 reflections
*c* = 11.2429 (13) Å	θ = 2.2–28.2º
α = 105.073 (4)º	µ = 0.33 mm^−^^1^
β = 94.105 (1)º	*T* = 295 K
γ = 90.815 (2)º	Block, colorless
*V* = 1099.2 (2) Å^3^	0.22 × 0.18 × 0.16 mm

### Data collection

**Table:**

Bruker SMART CCD diffractometer	3843 independent reflections
Radiation source: fine-focus sealed tube	3247 reflections with *I* > 2σ(*I*)
Monochromator: graphite	*R*_int_ = 0.015
*T* = 295 K	θ_max_ = 25.1º
φ and ω scans	θ_min_ = 1.9º
Absorption correction: multi-scan(SADABS; Sheldrick, 1996)	*h* = −10→10
*T*_min_ = 0.931, *T*_max_ = 0.949	*k* = −12→13
5721 measured reflections	*l* = −12→13

### Refinement

**Table:**

Refinement on *F*^2^	Secondary atom site location: difference Fourier map
Least-squares matrix: full	Hydrogen site location: inferred from neighbouring sites
*R*[*F*^2^ > 2σ(*F*^2^)] = 0.033	H-atom parameters constrained
*wR*(*F*^2^) = 0.096	*w* = 1/[σ^2^(*F*_o_^2^) + (0.0471*P*)^2^ + 0.3054*P*] where *P* = (*F*_o_^2^ + 2*F*_c_^2^)/3
*S* = 1.04	(Δ/σ)_max_ = 0.001
3843 reflections	Δρ_max_ = 0.19 e Å^−^^3^
289 parameters	Δρ_min_ = −0.20 e Å^−^^3^
Primary atom site location: structure-invariant direct methods	Extinction correction: none

### Special details

**Table:**

Geometry. All e.s.d.'s (except the e.s.d. in the dihedral angle between two l.s. planes) are estimated using the full covariance matrix. The cell e.s.d.'s are taken into account individually in the estimation of e.s.d.'s in distances, angles and torsion angles; correlations between e.s.d.'s in cell parameters are only used when they are defined by crystal symmetry. An approximate (isotropic) treatment of cell e.s.d.'s is used for estimating e.s.d.'s involving l.s. planes.
Refinement. Refinement of *F*^2^ against ALL reflections. The weighted *R*-factor *wR* and goodness of fit *S* are based on *F*^2^, conventional *R*-factors *R* are based on *F*, with *F* set to zero for negative *F*^2^. The threshold expression of *F*^2^ > σ(*F*^2^) is used only for calculating *R*-factors(gt) *etc*. and is not relevant to the choice of reflections for refinement. *R*-factors based on *F*^2^ are statistically about twice as large as those based on *F*, and *R*- factors based on ALL data will be even larger.

### Fractional atomic coordinates and isotropic or equivalent isotropic displacement parameters (Å^2^)

**Table:**

	*x*	*y*	*z*	*U*_iso_*/*U*_eq_	
Cl1	0.45143 (5)	0.67427 (5)	0.57622 (4)	0.05362 (15)	
Cl2	−0.04871 (6)	0.86602 (4)	0.57393 (5)	0.05615 (16)	
O1	0.52846 (13)	1.00138 (12)	0.17138 (11)	0.0470 (3)	
O2	0.74973 (16)	1.02523 (15)	0.27180 (14)	0.0658 (4)	
O3	0.03984 (14)	0.32842 (11)	0.17193 (12)	0.0491 (3)	
O4	0.25535 (16)	0.36297 (15)	0.28101 (15)	0.0665 (4)	
N1	0.60339 (17)	0.75273 (15)	0.11659 (13)	0.0479 (4)	
N2	0.11712 (16)	0.54988 (15)	0.11822 (13)	0.0459 (4)	
C1	0.6356 (2)	0.6329 (2)	0.09146 (18)	0.0565 (5)	
H1	0.6942	0.6023	0.0270	0.068*	
C2	0.5871 (2)	0.54890 (18)	0.15594 (19)	0.0566 (5)	
H2	0.6132	0.4652	0.1338	0.068*	
C3	0.5019 (2)	0.59019 (17)	0.25069 (17)	0.0478 (4)	
H3	0.4689	0.5351	0.2938	0.057*	
C4	0.46384 (17)	0.71804 (15)	0.28332 (15)	0.0376 (4)	
C5	0.37582 (18)	0.77105 (17)	0.38191 (15)	0.0400 (4)	
C6	0.3457 (2)	0.89608 (18)	0.40698 (17)	0.0472 (4)	
H6	0.2883	0.9305	0.4713	0.057*	
C7	0.3996 (2)	0.97351 (17)	0.33765 (17)	0.0471 (4)	
H7	0.3782	1.0584	0.3562	0.057*	
C8	0.48297 (18)	0.92409 (16)	0.24364 (15)	0.0393 (4)	
C9	0.51836 (17)	0.79539 (16)	0.21255 (15)	0.0374 (4)	
C10	0.3155 (2)	0.6929 (2)	0.45894 (18)	0.0502 (5)	
H10A	0.2836	0.6108	0.4065	0.060*	
H10B	0.2319	0.7332	0.4979	0.060*	
C11	0.6674 (2)	1.04787 (17)	0.19412 (17)	0.0462 (4)	
C12	0.6965 (3)	1.1301 (2)	0.1117 (2)	0.0681 (6)	
H12A	0.6399	1.2041	0.1332	0.102*	
H12B	0.6704	1.0851	0.0274	0.102*	
H12C	0.7979	1.1542	0.1213	0.102*	
C13	0.1446 (2)	0.6560 (2)	0.09004 (18)	0.0542 (5)	
H13	0.2051	0.6536	0.0268	0.065*	
C14	0.0896 (2)	0.7722 (2)	0.14814 (19)	0.0557 (5)	
H14	0.1119	0.8437	0.1226	0.067*	
C15	0.0033 (2)	0.77951 (17)	0.24219 (17)	0.0485 (4)	
H15	−0.0332	0.8563	0.2826	0.058*	
C16	−0.03079 (17)	0.66915 (16)	0.27844 (15)	0.0382 (4)	
C17	−0.11980 (18)	0.66651 (17)	0.37671 (16)	0.0418 (4)	
C18	−0.1492 (2)	0.55457 (19)	0.40301 (17)	0.0502 (5)	
H18	−0.2071	0.5533	0.4671	0.060*	
C19	−0.0938 (2)	0.44153 (18)	0.33514 (17)	0.0490 (4)	
H19	−0.1159	0.3661	0.3535	0.059*	
C20	−0.00775 (18)	0.44301 (16)	0.24263 (16)	0.0411 (4)	
C21	0.02808 (17)	0.55580 (16)	0.21112 (14)	0.0373 (4)	
C22	−0.1815 (2)	0.78404 (19)	0.45259 (18)	0.0527 (5)	
H22A	−0.2676	0.7626	0.4887	0.063*	
H22B	−0.2098	0.8385	0.4001	0.063*	
C23	0.1777 (2)	0.29749 (18)	0.19924 (18)	0.0472 (4)	
C24	0.2111 (3)	0.1743 (2)	0.1156 (2)	0.0684 (6)	
H24A	0.2125	0.1823	0.0326	0.103*	
H24B	0.1380	0.1123	0.1183	0.103*	
H24C	0.3044	0.1486	0.1417	0.103*	

### Atomic displacement parameters (Å^2^)

**Table:**

	*U*^11^	*U*^22^	*U*^33^	*U*^12^	*U*^13^	*U*^23^
Cl1	0.0594 (3)	0.0552 (3)	0.0514 (3)	−0.0014 (2)	−0.0012 (2)	0.0249 (2)
Cl2	0.0605 (3)	0.0447 (3)	0.0550 (3)	0.0013 (2)	0.0009 (2)	−0.0007 (2)
O1	0.0496 (7)	0.0482 (7)	0.0475 (7)	−0.0055 (6)	−0.0013 (6)	0.0223 (6)
O2	0.0525 (8)	0.0773 (10)	0.0691 (9)	−0.0129 (7)	−0.0105 (7)	0.0268 (8)
O3	0.0510 (7)	0.0381 (7)	0.0513 (7)	0.0055 (5)	−0.0045 (6)	0.0015 (5)
O4	0.0501 (8)	0.0702 (10)	0.0749 (10)	0.0054 (7)	−0.0100 (7)	0.0154 (8)
N1	0.0495 (9)	0.0516 (9)	0.0409 (8)	0.0002 (7)	0.0078 (7)	0.0081 (7)
N2	0.0447 (8)	0.0536 (9)	0.0374 (8)	0.0020 (7)	0.0028 (6)	0.0082 (7)
C1	0.0581 (12)	0.0583 (13)	0.0464 (11)	0.0065 (10)	0.0095 (9)	0.0000 (9)
C2	0.0626 (13)	0.0410 (10)	0.0609 (12)	0.0075 (9)	0.0013 (10)	0.0043 (9)
C3	0.0507 (11)	0.0403 (10)	0.0521 (11)	−0.0017 (8)	−0.0036 (9)	0.0140 (8)
C4	0.0341 (8)	0.0393 (9)	0.0383 (8)	−0.0027 (7)	−0.0046 (7)	0.0105 (7)
C5	0.0329 (9)	0.0483 (10)	0.0406 (9)	−0.0023 (7)	−0.0018 (7)	0.0161 (8)
C6	0.0464 (10)	0.0529 (11)	0.0434 (10)	0.0076 (8)	0.0101 (8)	0.0124 (8)
C7	0.0525 (11)	0.0387 (9)	0.0500 (10)	0.0065 (8)	0.0045 (8)	0.0110 (8)
C8	0.0389 (9)	0.0414 (9)	0.0390 (9)	−0.0044 (7)	−0.0031 (7)	0.0150 (7)
C9	0.0345 (9)	0.0417 (9)	0.0344 (8)	−0.0029 (7)	−0.0029 (7)	0.0090 (7)
C10	0.0404 (10)	0.0624 (12)	0.0529 (11)	−0.0043 (8)	0.0020 (8)	0.0247 (9)
C11	0.0500 (11)	0.0407 (10)	0.0449 (10)	−0.0059 (8)	0.0058 (9)	0.0054 (8)
C12	0.0832 (16)	0.0607 (13)	0.0636 (13)	−0.0206 (11)	0.0130 (12)	0.0212 (11)
C13	0.0519 (11)	0.0681 (14)	0.0444 (10)	−0.0022 (10)	0.0078 (9)	0.0171 (9)
C14	0.0616 (12)	0.0541 (12)	0.0553 (12)	−0.0078 (9)	0.0003 (10)	0.0231 (10)
C15	0.0516 (11)	0.0388 (10)	0.0530 (11)	0.0007 (8)	−0.0022 (9)	0.0103 (8)
C16	0.0338 (8)	0.0393 (9)	0.0379 (9)	−0.0004 (7)	−0.0052 (7)	0.0060 (7)
C17	0.0352 (9)	0.0435 (10)	0.0423 (9)	0.0017 (7)	−0.0002 (7)	0.0044 (7)
C18	0.0477 (11)	0.0554 (11)	0.0463 (10)	−0.0038 (8)	0.0102 (8)	0.0095 (9)
C19	0.0541 (11)	0.0433 (10)	0.0509 (10)	−0.0041 (8)	0.0037 (9)	0.0149 (8)
C20	0.0403 (9)	0.0363 (9)	0.0420 (9)	0.0025 (7)	−0.0052 (7)	0.0041 (7)
C21	0.0315 (8)	0.0426 (9)	0.0347 (8)	0.0008 (7)	−0.0042 (7)	0.0062 (7)
C22	0.0436 (10)	0.0556 (12)	0.0526 (11)	0.0065 (8)	0.0040 (8)	0.0022 (9)
C23	0.0489 (11)	0.0473 (10)	0.0504 (11)	0.0072 (8)	0.0062 (9)	0.0207 (9)
C24	0.0816 (16)	0.0563 (13)	0.0728 (14)	0.0253 (11)	0.0222 (12)	0.0209 (11)

### Geometric parameters (Å, °)

**Table:**

Cl1—C10	1.8072 (19)	C10—H10A	0.970
Cl2—C22	1.807 (2)	C10—H10B	0.970
O1—C11	1.356 (2)	C11—C12	1.488 (3)
O1—C8	1.399 (2)	C12—H12A	0.960
O2—C11	1.194 (2)	C12—H12B	0.960
O3—C23	1.357 (2)	C12—H12C	0.960
O3—C20	1.400 (2)	C13—C14	1.397 (3)
O4—C23	1.193 (2)	C13—H13	0.930
N1—C1	1.318 (3)	C14—C15	1.355 (3)
N1—C9	1.367 (2)	C14—H14	0.930
N2—C13	1.313 (3)	C15—C16	1.416 (3)
N2—C21	1.364 (2)	C15—H15	0.930
C1—C2	1.402 (3)	C16—C21	1.418 (2)
C1—H1	0.930	C16—C17	1.429 (2)
C2—C3	1.356 (3)	C17—C18	1.367 (3)
C2—H2	0.930	C17—C22	1.497 (2)
C3—C4	1.415 (2)	C18—C19	1.404 (3)
C3—H3	0.930	C18—H18	0.930
C4—C9	1.417 (2)	C19—C20	1.356 (3)
C4—C5	1.427 (2)	C19—H19	0.930
C5—C6	1.367 (3)	C20—C21	1.417 (2)
C5—C10	1.497 (2)	C22—H22A	0.970
C6—C7	1.404 (3)	C22—H22B	0.970
C6—H6	0.930	C23—C24	1.486 (3)
C7—C8	1.354 (2)	C24—H24A	0.960
C7—H7	0.930	C24—H24B	0.960
C8—C9	1.417 (2)	C24—H24C	0.960
			
C11—O1—C8	117.15 (14)	H12A—C12—H12C	109.5
C23—O3—C20	117.00 (14)	H12B—C12—H12C	109.5
C1—N1—C9	116.68 (16)	N2—C13—C14	124.74 (18)
C13—N2—C21	116.52 (16)	N2—C13—H13	117.6
N1—C1—C2	123.99 (18)	C14—C13—H13	117.6
N1—C1—H1	118.0	C15—C14—C13	119.15 (19)
C2—C1—H1	118.0	C15—C14—H14	120.4
C3—C2—C1	119.73 (18)	C13—C14—H14	120.4
C3—C2—H2	120.1	C14—C15—C16	119.46 (18)
C1—C2—H2	120.1	C14—C15—H15	120.3
C2—C3—C4	119.31 (18)	C16—C15—H15	120.3
C2—C3—H3	120.3	C15—C16—C21	116.61 (16)
C4—C3—H3	120.3	C15—C16—C17	123.98 (16)
C9—C4—C3	116.66 (16)	C21—C16—C17	119.42 (16)
C9—C4—C5	119.63 (15)	C18—C17—C16	119.63 (16)
C3—C4—C5	123.71 (16)	C18—C17—C22	118.87 (17)
C6—C5—C4	119.42 (16)	C16—C17—C22	121.50 (17)
C6—C5—C10	119.06 (16)	C17—C18—C19	121.30 (17)
C4—C5—C10	121.51 (16)	C17—C18—H18	119.4
C5—C6—C7	121.35 (16)	C19—C18—H18	119.4
C5—C6—H6	119.3	C20—C19—C18	119.64 (18)
C7—C6—H6	119.3	C20—C19—H19	120.2
C8—C7—C6	119.72 (17)	C18—C19—H19	120.2
C8—C7—H7	120.1	C19—C20—O3	118.62 (16)
C6—C7—H7	120.1	C19—C20—C21	122.02 (16)
C7—C8—O1	118.65 (16)	O3—C20—C21	119.26 (15)
C7—C8—C9	121.91 (16)	N2—C21—C20	118.54 (15)
O1—C8—C9	119.34 (15)	N2—C21—C16	123.49 (16)
N1—C9—C4	123.63 (16)	C20—C21—C16	117.98 (15)
N1—C9—C8	118.41 (15)	C17—C22—Cl2	110.29 (13)
C4—C9—C8	117.96 (15)	C17—C22—H22A	109.6
C5—C10—Cl1	110.54 (12)	Cl2—C22—H22A	109.6
C5—C10—H10A	109.5	C17—C22—H22B	109.6
Cl1—C10—H10A	109.5	Cl2—C22—H22B	109.6
C5—C10—H10B	109.5	H22A—C22—H22B	108.1
Cl1—C10—H10B	109.5	O4—C23—O3	122.23 (17)
H10A—C10—H10B	108.1	O4—C23—C24	127.61 (19)
O2—C11—O1	122.74 (17)	O3—C23—C24	110.15 (18)
O2—C11—C12	127.10 (19)	C23—C24—H24A	109.5
O1—C11—C12	110.15 (17)	C23—C24—H24B	109.5
C11—C12—H12A	109.5	H24A—C24—H24B	109.5
C11—C12—H12B	109.5	C23—C24—H24C	109.5
H12A—C12—H12B	109.5	H24A—C24—H24C	109.5
C11—C12—H12C	109.5	H24B—C24—H24C	109.5
			
C9—N1—C1—C2	0.6 (3)	C21—N2—C13—C14	0.0 (3)
N1—C1—C2—C3	−0.2 (3)	N2—C13—C14—C15	1.2 (3)
C1—C2—C3—C4	−0.3 (3)	C13—C14—C15—C16	−0.8 (3)
C2—C3—C4—C9	0.2 (2)	C14—C15—C16—C21	−0.7 (2)
C2—C3—C4—C5	−179.53 (17)	C14—C15—C16—C17	179.58 (17)
C9—C4—C5—C6	−0.2 (2)	C15—C16—C17—C18	178.55 (17)
C3—C4—C5—C6	179.55 (16)	C21—C16—C17—C18	−1.2 (2)
C9—C4—C5—C10	179.84 (15)	C15—C16—C17—C22	−1.8 (3)
C3—C4—C5—C10	−0.4 (2)	C21—C16—C17—C22	178.48 (15)
C4—C5—C6—C7	0.0 (3)	C16—C17—C18—C19	−0.1 (3)
C10—C5—C6—C7	179.96 (17)	C22—C17—C18—C19	−179.78 (17)
C5—C6—C7—C8	0.2 (3)	C17—C18—C19—C20	0.9 (3)
C6—C7—C8—O1	176.36 (15)	C18—C19—C20—O3	−176.61 (16)
C6—C7—C8—C9	−0.1 (3)	C18—C19—C20—C21	−0.3 (3)
C11—O1—C8—C7	102.11 (19)	C23—O3—C20—C19	−100.93 (19)
C11—O1—C8—C9	−81.32 (19)	C23—O3—C20—C21	82.69 (19)
C1—N1—C9—C4	−0.6 (3)	C13—N2—C21—C20	178.39 (16)
C1—N1—C9—C8	179.11 (16)	C13—N2—C21—C16	−1.7 (2)
C3—C4—C9—N1	0.2 (2)	C19—C20—C21—N2	178.97 (16)
C5—C4—C9—N1	180.00 (15)	O3—C20—C21—N2	−4.8 (2)
C3—C4—C9—C8	−179.53 (15)	C19—C20—C21—C16	−0.9 (2)
C5—C4—C9—C8	0.3 (2)	O3—C20—C21—C16	175.30 (14)
C7—C8—C9—N1	−179.84 (16)	C15—C16—C21—N2	2.0 (2)
O1—C8—C9—N1	3.7 (2)	C17—C16—C21—N2	−178.21 (15)
C7—C8—C9—C4	−0.1 (2)	C15—C16—C21—C20	−178.08 (15)
O1—C8—C9—C4	−176.54 (14)	C17—C16—C21—C20	1.7 (2)
C6—C5—C10—Cl1	−99.09 (17)	C18—C17—C22—Cl2	96.61 (18)
C4—C5—C10—Cl1	80.86 (18)	C16—C17—C22—Cl2	−83.08 (19)
C8—O1—C11—O2	0.1 (3)	C20—O3—C23—O4	1.3 (3)
C8—O1—C11—C12	−178.64 (16)	C20—O3—C23—C24	−179.61 (16)
